# Supplementary material for: Adipose Tissue Caveolin-1 Upregulation in Obesity Involves TNF-α/NF-κB Mediated Signaling
Source: Cells. 2023 Mar 27;12(7):1019. doi: 10.3390/cells12071019 (PMC10093236; doi:10.3390/cells12071019)
Supplement: Supplementary file 1 [file cells-12-01019-s001.zip › cells-2091670-supplementary.pdf]

Supplementary Materials

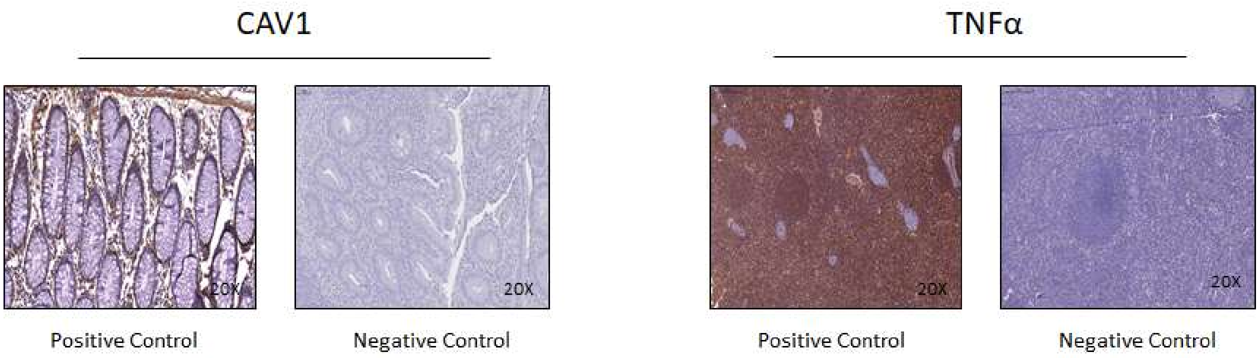

Figure S1. Caveolin-1 (CAV1) and TNF-α antibodies specificity validation using spleen tissues.

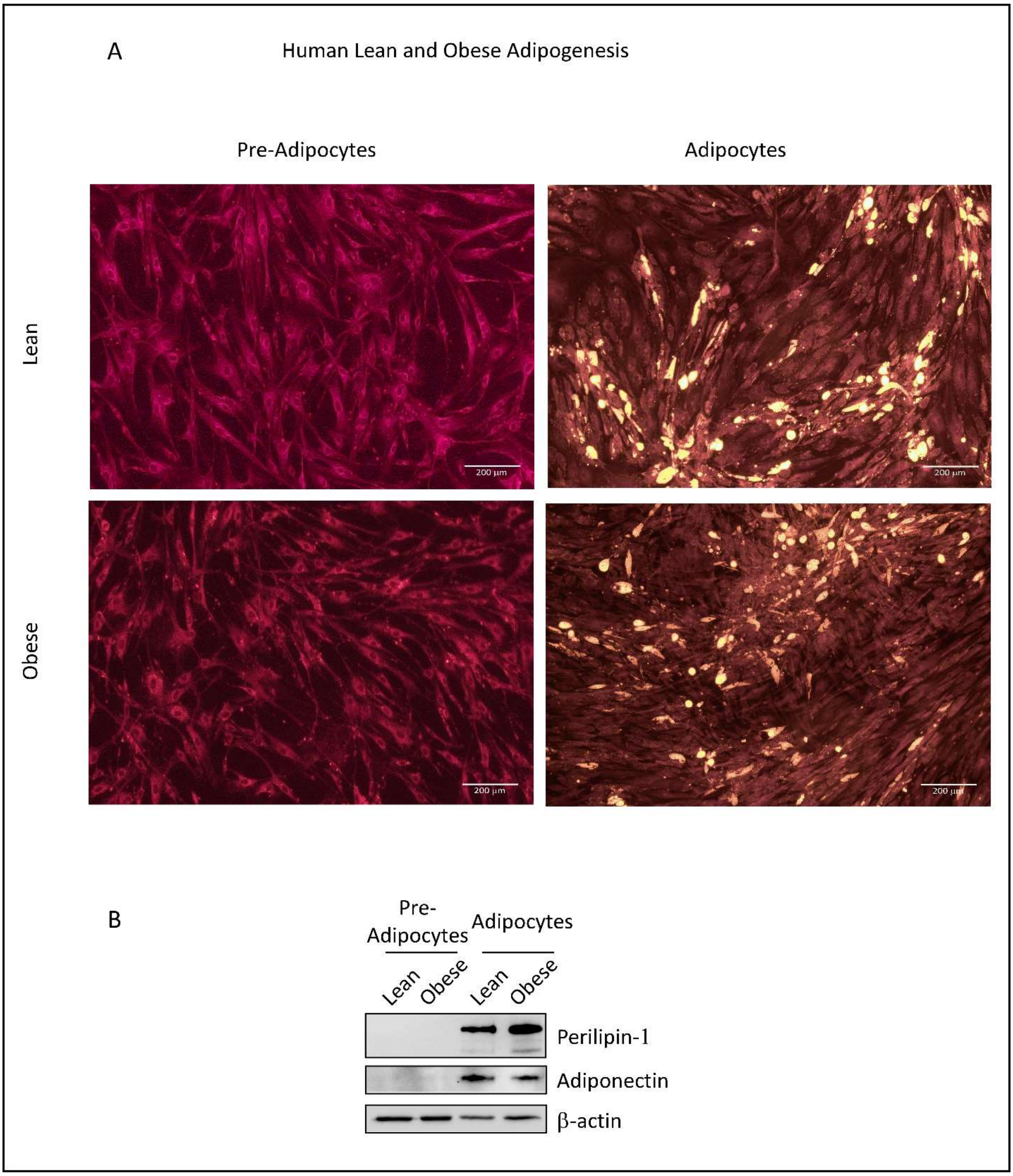

Figure S2. Human pre-adipocytes were differentiated into adipocytes. (**A**) Adipocytes were monitored for lipid droplet appearance in the cytoplasm using Nile Red staining (Magnification 100×). (**B**) The differentiation authenticity was detected by Western blot assays using antibodies against the adipocyte's markers.

Table S1. List of TaqMan Assays used for RT-PCR.

Primer List	Assay ID	Primer List	Assay ID
IL-2	Hs00174114_m1	CXCL9	Hs00171065_m1
IL-5	Hs01548712_g1	CXCL10	Hs01124251_g1
IL-6	Hs00985639_m1	CXCL11	Hs04187682_g1
IL-8	Hs00174103_m1	TLR2	Hs01822448_m1
IL-10	Hs00961622_m1	TLR3	Hs01551078_m1
IL-12A	Hs01073447_m1	TLR4	Hs00152939_m1
IL-13	Hs00174379_m1	TLR7	Hs01933259_s1
IL23A	Hs00900828_g1	TLR8	Hs07292888_s1
IL1RL1	Hs00545033_m1	TLR9	Hs00370913_s1
IL2RA	Hs00907779_m1	TLR10	Hs01935337_s1
CCL2	Hs00234140_m1	IRF4	Hs00180031_m1
CCL3	Hs04194942_s1	CD16	Hs04334165_m1
CCL8	Hs04187715_m1	CD68	Hs02836816_g1
CCL15	Hs00361122_m1	CD86	Hs01567026_m1
CCL18	Hs00268113_m1	CD163	Hs00174705_m1
CCR1	Hs00928897_s1	NF-κB	Hs00765730_m1
CCR2	Hs00704702_s1	TNF-α	Hs01113624_g1
GAPDH	Hs03929097_g1	Caveolin 1	Hs00971716_m1
Mouse Caveolin-1	Mm00483057_m1		
